# Effectiveness of azvudine in reducing mortality of COVID-19 patients: a systematic review and meta-analysis

**DOI:** 10.1186/s12985-024-02316-y

**Published:** 2024-02-23

**Authors:** Yaqi Wang, Huaiya Xie, Luo Wang, Junping Fan, Ying Zhang, Siqi Pan, Wangji Zhou, Qiaoling Chen, Xueqi Liu, Aohua Wu, Hong Zhang, Jinglan Wang, Xinlun Tian

**Affiliations:** 1 Department of Pulmonary and Critical Care Medicine, Peking Union Medical College Hospital, Chinese Academy of Medical Sciences & Peking Union Medical College, No.1 Shuaifuyuan, Wangfujing, Dongcheng District, 100730 Beijing, China; 2 International Medical Services, Peking Union Medical College Hospital, Chinese Academy of Medical Sciences & Peking Union Medical College, Beijing, China

**Keywords:** Azvudine, COVID-19, SARS-CoV-2, Mortality, Adverse event

## Abstract

**Background:**

Azvudine has been approved for the treatment of coronavirus disease 2019 (COVID-19) patients in China, and this meta-analysis aims to illustrate the safety of azvudine and its effectiveness in reducing mortality.

**Methods:**

PubMed, Embase, Web of science, Cochrane Library and the Epistemonikos COVID-19 Living Overview of Evidence database (L.OVE) were searched to aggregate currently published studies. Cochrane risk of bias tool and ROBINS-I tool were used to assess the risk of bias of randomized controlled study and cohort study respectively. Odds radios (ORs) with 95% confidence interval (CIs) were combined for dichotomous variables. Publication bias was assessed by Egger’s test and funnel plots.

**Results:**

A total of 184 articles were retrieved from the included databases and 17 studies were included into the final analysis. Pooled analysis showed that azvudine significantly reduced mortality risk in COVID-19 patients compared with controls (OR: 0.41, 95%CI 0.31–0.54, *p* < 0.001). Besides, either mild to moderate or severe COVID-19 patients could benefit from azvudine administration. There was no significant difference in the incidence of ICU admission (OR: 0.90, 95%CI 0.47–1.72, *p* = 0.74) and invasive ventilation (OR: 0.94, 95%CI 0.54–1.62, *p* = 0.82) between azvudine and control group. The incidence of adverse events was similar between azvudine and control (OR: 1.26, 95%CI 0.59–2.70, *p* = 0.56).

**Conclusions:**

This meta-analysis suggests that azvudine could reduce the mortality risk of COVID-19 patients, and the safety of administration is acceptable.

**Trial registration:**

PROSPERO; No.: CRD42023462988; URL: https://www.crd.york.ac.uk/prospero/.

**Supplementary Information:**

The online version contains supplementary material available at 10.1186/s12985-024-02316-y.

## Background

Since the outbreak of the severe acute respiratory syndrome coronavirus 2 (SARS-CoV-2) at the end of 2019, more than 700 million people have been infected, and nearly 7 million people have died due to the viral infection as of September 9, 2023, which has caused a serious burden on the public and the economy [1, 2]. During the Omicron variant of SARS-CoV-2 epidemic in China at the end of 2022, a large number of patients emerged in less than 2 months, which called for an urgent need for effective, safe and economical antiviral drugs [3, 4]. Besides Nirmatrelvir/Ritonavir (Paxlovid), Molnupiravir and Remdesivir, Azvudine, a nucleotide analogue, has gained emergency approval for treatment of coronavirus disease 2019 (COVID-19) patients in China since September, 2022 [5, 6].

Azvudine can block the RNA replication of the virus through inhibiting RNA-dependent RNA polymerase, and has been shown to be effective against SARS-CoV-2 [7, 8]. Several studies exploring azvudine treatment for COVID-19 patients had reported different clinical outcomes and adverse events, without conclusive results [9–13]. Besides, there have been retrospective studies comparing the efficacy of azvudine with Paxlovid in the treatment of patients with COVID-19, and the results remain controversial [14, 15]. There was a published meta-analysis illustrating the efficacy of azvudine on shortening time to nucleic-acid negative conversion and its safety [16]. However, quantitative pooled analyses were lack, and other clinical outcomes were not discussed. We thus conducted this meta-analysis, to comprehensively clarify its antiviral efficacy, and safety of azvudine for SARS-CoV-2 in details.

## Methods

We conducted and reported this meta-analysis according to the Preferred Reporting Items for Systematic Reviews and Meta-Analyses guidelines [17]. The protocol was registered with PROSPERO (CRD42023462988).

### Search strategy and study identification

We performed a systematic literature search using the online databases: PubMed, Embase, Web of sciences, Cochrane Library and the Epistemonikos COVID-19 Living Overview of Evidence (L.OVE) database. All publications before August 23, 2022, were identified using the following keywords: “COVID-19”, “SARS-CoV-2”, “Azvudine” and “FNC”. We also manually reviewed the reference lists of relevant articles to identify additional studies.

Studies eligible for this meta-analysis met the following selection criteria: (1) randomized controlled studies (RCTs) or cohort studies investigating patients confirmed coronavirus-19 disease (COVID-19); (2) Azvudine as the treatment intervention with or without control; (3) efficacy and safety outcomes of interest; (4) studies written in English. Reviews, case reports, case series were excluded. Conference abstracts reporting similar results and conducted by the same research group were superseded by publications.

### Study selection, data extraction, and risk of bias assessment

Two reviewers (Yaqi Wang and Huaiya Xie) independently performed eligibility evaluation, data extraction, and risk of bias assessment. Disagreements between the two reviewers were resolved through discussion until a consensus was reached. We extracted data on general information (first author, publication year, country, study design), participants (sample size, age, and sex), antiviral therapies specific to COVID-19, efficacy and safety outcomes (mortality risks showed with Hazards ratios (HRs) or odds ratios (ORs) with 95% confidence intervals (95% CIs), time to first nucleic-acid negative conversion, intensive care unit (ICU) admission, rate of progression to invasive mechanical ventilation (IMV) or extracorporeal membrane oxygenation (ECMO), and incidence of any AEs. We preferred results using propensity score matching for cohort studies; otherwise, data from total sample were extracted. Moreover, we also did further analyses for patients over 65 years old, with different severity, and with comorbidities (hypertension, diabetes mellitus, and cardiovascular diseases).

We employed Cochrane risk of bias tool to evaluate methodological quality of RCTs [18], and assessed risk of bias in nonrandomized studies using ROBINS-I tool [19].

### Statistics

We presented combined results as ORs with 95% CIs for dichotomous variables. We measured heterogeneity between studies using Higgins’s test. A random-effects model for pooled quantitative analysis was applied when high heterogeneity was identified (i.e., *I*^*2*^ statistic > 50%); otherwise, a fixed-effects model was used. Sensitivity analyses of adverse events were conducted based on study types to confirm the reliability of pooled analyses. We used Egger’s test and funnel plots to assess publication bias (Supplementary Figs. 1–2 [Additional file 1]). Statistical significance was set at *p* < 0.05. All statistical analyses were performed using R (version 4.2.2, R Foundation for Statistical Computing, Vienna, Austria).

## Results

### Progress of selection and characteristics of study

**Fig. 1:**
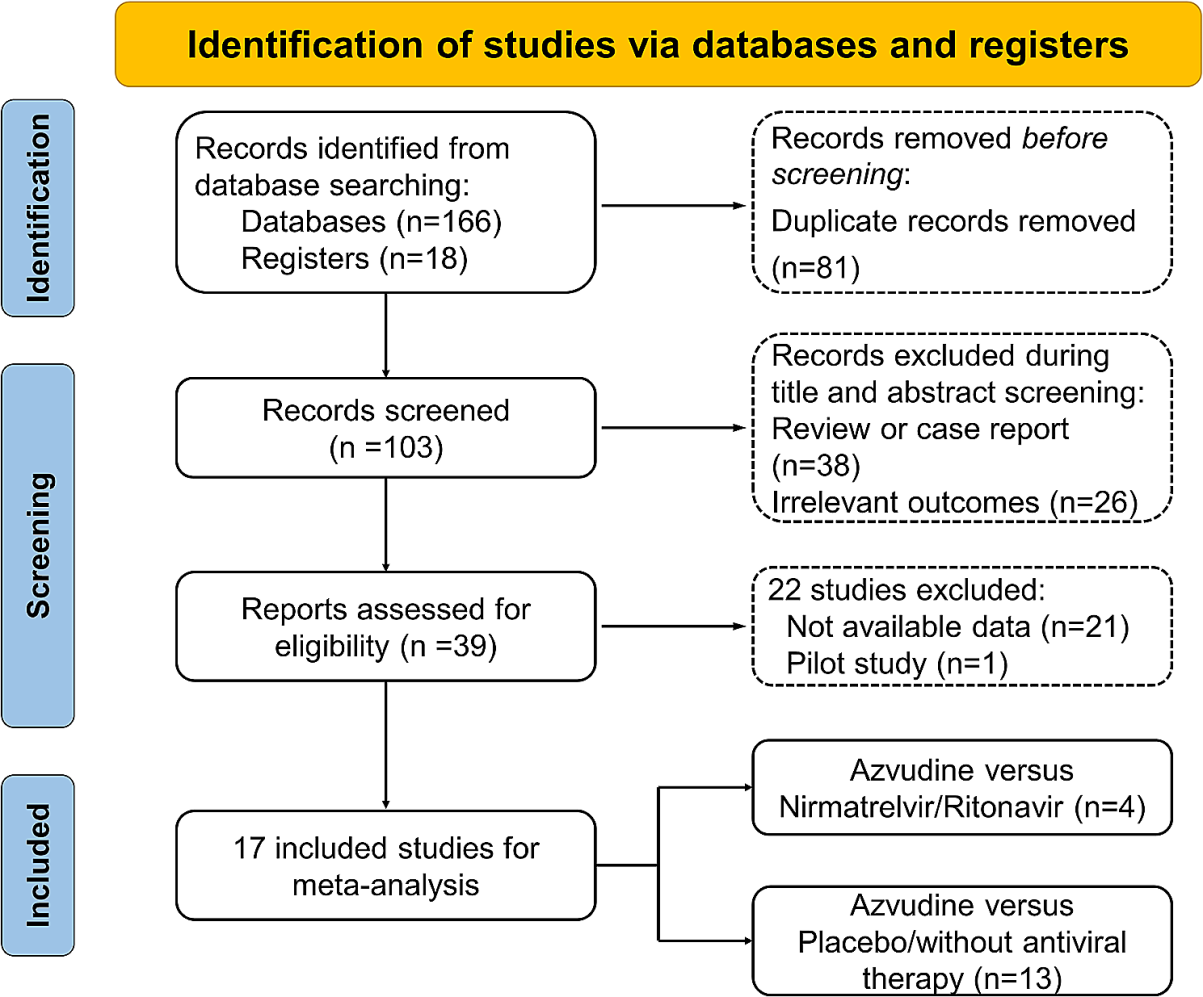
The searching, screening and filtering process of studies

A total of 184 articles were retrieved from the included databases (Supplementary Table 1 [Additional file 1]), and we finally included 7746 patients from 17 studies after selection (Fig. 1). The main characteristics of the study were summarized in Table 1 [9–15, 20–29]. We in total enrolled 3 RCTs, and 14 retrospective cohort studies. Among them, 4 studies compared the efficacy and safety of azvudine and Paxlovid in fighting against COVID-19, while remaining 13 studies compared azvudine with placebo, or no specific antiviral therapies. Except two RCTs conducted in Brazil, other studies were progressed and reported in China. We employed Cochrane risk of bias tool for RCTs, and ROBIN-I tools for cohort studies to evaluate bias of studies. Most studies were classified into low or moderated bias, except one RCT conducted by Ren et al. [9] without blinding, one study with only abstract and unclear information [28] and another retrospective study conducted by Shao et. al [21] without confounding adjustment. All aforementioned results were showed in Supplementary Tables 2–3 [Additional file 1], and illustrated in Supplementary Fig. 3 [Additional file 1] as well.

**Table 1:** Baseline characteristics of the included studies

First author	Publication year	Study type	No. of patients	Country	Antiviral therapy	Outcomes	Risk
Zhigang Ren	2020	RCT	20	China	FNC vs No antiviral therapy	*T*_FNANC_, AEs	High
Renato Martins da Silva	2023	RCT	312	Brazil	FNC vs No antiviral therapy	*T*_FNANC_, AEs	Low
Paula Cabral	2022	RCT	180	Brazil	FNC vs No antiviral therapy	*T*_FNANC_, AEs	Low
Kaican Zong	2023	R	585	China	FNC vs No antiviral therapy	Mortality	Moderate
Minxue Shen	2023	R	452	China	FNC vs No antiviral therapy	Mortality, ICU admission, IMV/ECMO	Moderate
Guangtong Deng	2023	R	562	China	FNC vs RN	Mortality, ICU admission, IMV/ECMO	Moderate
Qinqin Zhao	2023	R	286	China	FNC vs RN	Mortality, *T*_FNANC_, AEs	Moderate
Hui Yang	2023	R	804	China	FNC vs No antiviral therapy	AEs	Moderate
Jiasheng Shao	2023	R	686	China	FNC vs No antiviral therapy	Mortality	Serious
Ru Chen	2023	R	198	China	FNC vs No antiviral therapy	Mortality, ICU admission, IMV/ECMO	Moderate
Shunlai Shang	2023	R	364	China	FNC vs No antiviral therapy	AEs	Moderate
Wenmei Chen	2023	R	207	China	FNC vs No antiviral therapy	*T*_FNANC_, AEs	Moderate
Xinjie Han	2023	R	856	China	FNC vs No antiviral therapy	Mortality	Moderate
Yating Dian	2023	R	456	China	FNC vs RN	Mortality, ICU admission, IMV/ECMO	Moderate
Yuan Gao	2023	R	134	China	FNC vs RN	*T*_FNANC_	Moderate
Yiling Zhou	2023	R	1154	China	FNC vs No antiviral therapy	Mortality	Unclear
Yuming Sun	2023	R	490	China	FNC vs No antiviral therapy	Mortality, ICU admission, IMV/ECMO	Moderate

RCT = randomized controlled study; R = retrospective cohort; FNC = azvudine; RN = Ritonavir-Nirmatrelvir; *T*_FNANC_ = time to first nucleic-acid negative conversion; AE = adverse event; ICU = intensive care unit; IMV = invasive mechanical ventilation; ECMO = extracorporeal membrane oxygenation

### The efficacy of azvudine

We collected four clinical outcomes for efficacy evaluation, including mortality, nucleic acid negative conversion time, ICU admission, and progression to IMV/ECMO.

#### Mortality

**Fig. 2:**
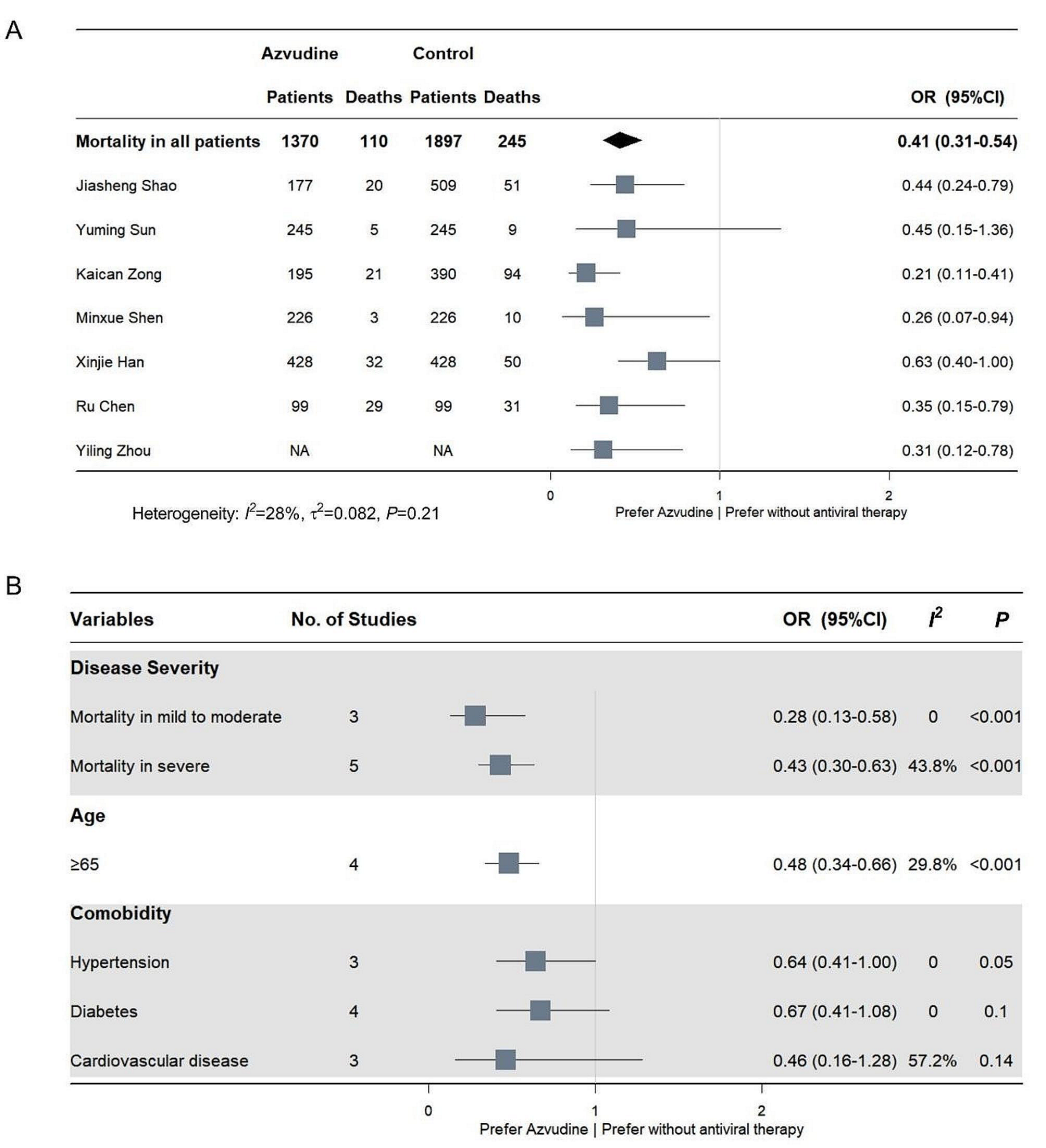
Effectiveness of azvudine on mortality. (**A**) Effectiveness of azvudine on mortality in COVID-19 patients. (**B**) Effectiveness of azvudine on mortality in different types of COVID-19 patients. NA = Not available

A pooled analysis of 7 studies involving 4421 patients showed that azvudine significantly reduced mortality in patients with COVID-19 compared with no antiviral drugs (OR: 0.41, 95%CI 0.31–0.54, *p* < 0.001) (Table 2; Fig. 2a). We further repeated the analysis in COVID-19 patients with special characteristics. Three and five studies, respectively, compared mortality risks between azvudine and no antiviral therapies, in patents of mild to moderate state, and severe form of COVID-19. Pooled analyses showed azvudine were still associated with decreased mortality risks no matter the disease severity (mild to moderate: OR 0.28, 95%CI 0.13–0.58, *p* < 0.001; severe: OR 0.43, 95%CI 0.30–0.63, *p* < 0.001) (Fig. 2b, Supplementary Fig. 4 [Additional file 1]). Pooled analysis of 4 studies showed azvudine associated with a 52% reduction of mortality risks in COVID-19 patients over 65 years old, compared with no antiviral therapies (OR 0.48, 95%CI 0.34–0.66, *p* < 0.001). Meanwhile, another 2 studies did not identify such associations in patients no more than 65 years old [12, 25]. However, similar relationships were not showed in patients with hypertension, diabetes mellitus, or cardiovascular diseases (Table 2; Fig. 2b).

**Table 2:** Meta-analysis results of relationships between different antiviral strategies and mortality risks

FNC vs No antiviral therapy	No. of cohorts	Effects model	*I*^2^ (%)	OR (95%CI)	*P*	Relationships	Publication bias (*P* value of Egger’s test)
Overall patients	7	Fixed	28	0.41 (0.31–0.54)	< 0.001	Decreased risk	0.20
Patients with mild to moderate COVID-19	3	Fixed	0	0.28 (0.13–0.58)	< 0.001	Decreased risk	0.52
Patients with severe COVID-19	5	Fixed	44	0.43 (0.30–0.63)	< 0.001	Decreased risk	0.22
Patients over 65 years old	4	Fixed	30	0.48 (0.34–0.66)	< 0.001	Decreased risk	0.83
Patients with hypertension	3	Fixed	0	0.64 (0.41-1.00)	0.05	No significance	0.51
Patients with diabetes	4	Fixed	0	0.67 (0.41–1.08)	0.10	No significance	0.72
Patients with cardiovascular disease	3	Random	57	0.46 (0.16–1.28)	0.1	No significance	< 0.001

FNC = azvudine; COVID-19 = Coronavirus Disease 2019

Two studies compared the efficacy of azvudine and Paxlovid on mortality risks of COVID-19 patients. A study conducted by Deng et al. [14] showed a lower mortality risk in azvudine (OR 0.38, 95%CI 0.15–0.98, *p* = 0.04), while another retrospective study found no significant difference between azvudine and Paxlovid (OR 1.27, 95%CI 0.47–3.42, *p* = 0.63) [15]. Besides, another study only contrasted mortality rate between 2 groups, which also showed mortality rates were comparable between the two groups (azvudine *n* = 4 vs. Paxlovid *n* = 11, *p* = 0.18) [26].

#### Nucleic acid negative conversion time

Three RCTs and one retrospective study reported time to first nucleic-acid negative conversion of COVID-19 patients using azvudine compared to controls, all demonstrating that azvudine significantly shortened the time to nucleic acid conversion [9–11, 24]. Another two studies compared the nucleic acid negative conversion time of COVID-19 patients receiving either azvudine and Paxlovid. The two articles both reported longer nucleic acid negative conversion time in azvudine group (median time 10 days vs. 5.8 days, and 16.5 days vs. 13 days, respectively) [15, 27].

#### ICU admission and progression to IMV/ECMO

**Fig. 3:**
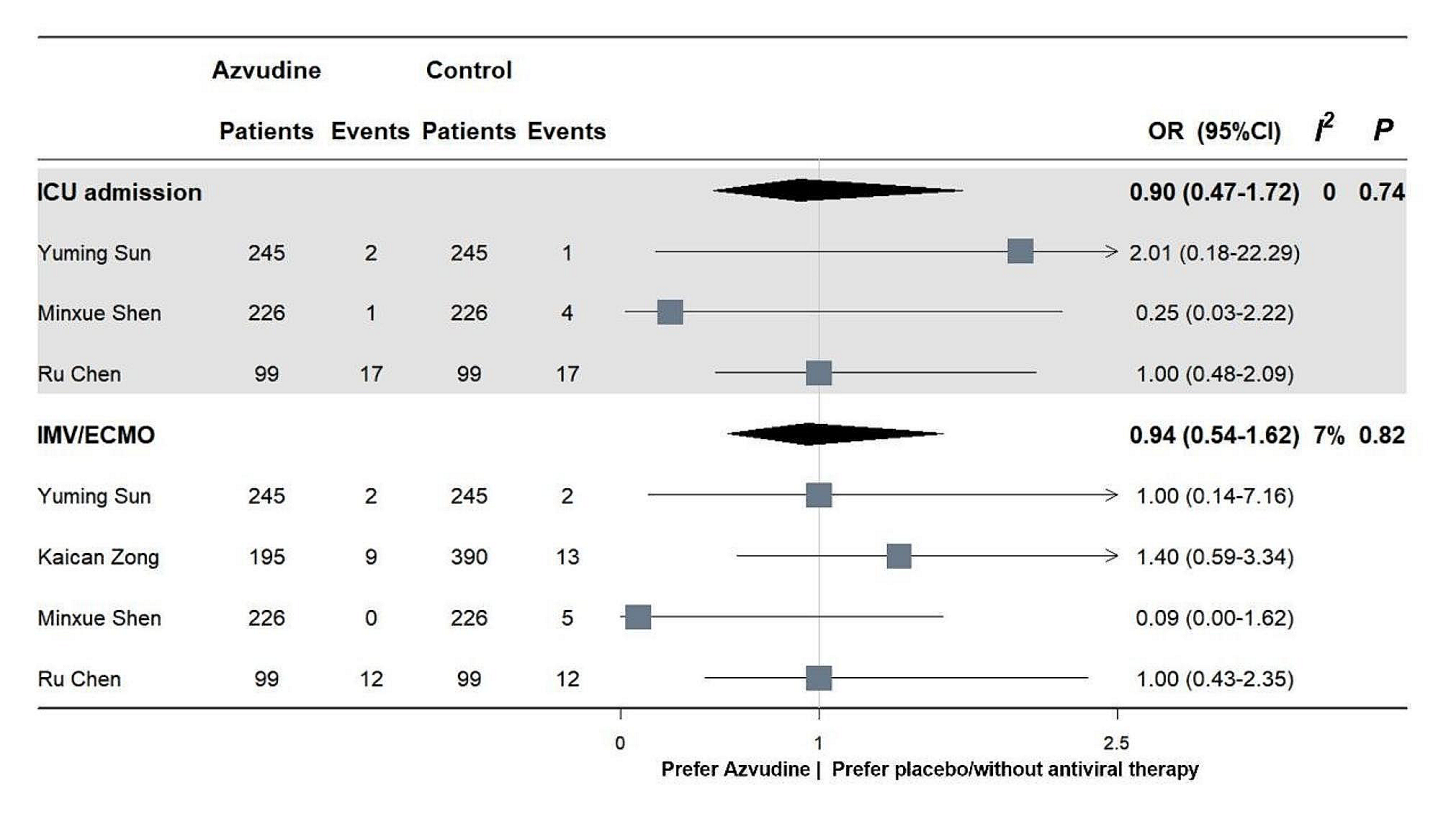
Effectiveness of azvudine on ICU admission and invasive ventilation in COVID-19 patients. ICU = intensive care unit; IMV = invasive mechanical ventilation; ECMO = extracorporeal membrane oxygenation

Pooled results of three retrospective cohort studies enrolling 570 patients did not find benefit on ICU admission rates in COVID-19 patients receiving azvudine, compared with those with no antiviral therapy (OR: 0.90, 95%CI 0.47–1.72, *p* = 0.74). Similarly, a pooled analysis of four studies showed no significant difference between two groups as for the incidence of IMV/ECMO (OR 0.94, 95%CI 0.54–1.62, *p* = 0.82). The results were also illustrated in Fig. 3.

### Safety evaluation of Azvudine

A total of six studies reported 497 adverse events (AEs). Relevant AEs could be classified into gastrointestinal symptoms, elevation of liver enzymes or serum creatinine, neurological symptoms, chest discomfort, rash, and decline of leukocytes or platelet.

**Fig. 4:**
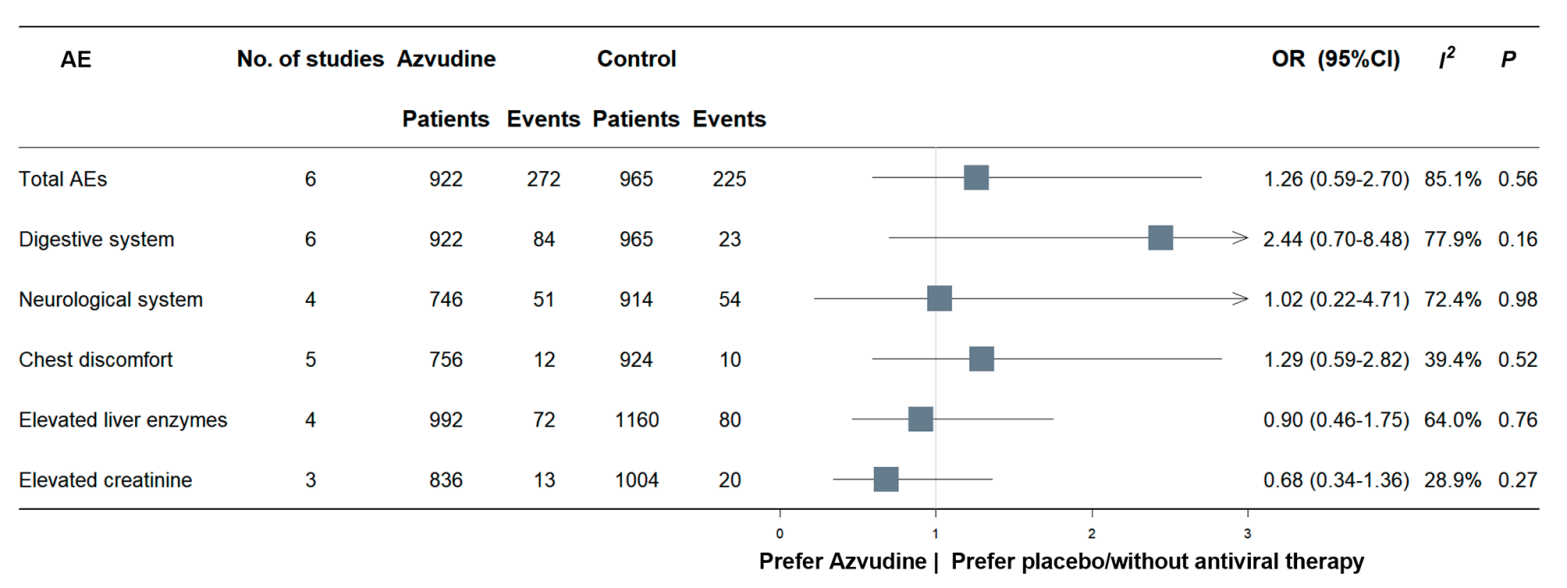
Incidence of adverse events of azvudine compared with controls. AE = adverse event

We employed random-effect model (*I*^*2*^ 85.1%, *p* < 0.001) for pooled analysis, and found no significant difference in the incidence of adverse events between using azvudine and no antiviral therapy (OR 1.26, 95%CI 0.59–2.70, *p* = 0.56). In addition, the incidences of gastrointestinal symptoms (OR: 2.44, 95%CI 0.70–8.48, *p* = 0.16), elevated liver enzymes (OR: 0.90, 95%CI 0.46–1.75, *p* = 0.76), elevated creatinine (OR: 0.68, 95%CI 0.34–1.36, *p* = 0.27), neurological symptoms (OR: 1.02, 95%CI 0.22–4.71, *p* = 0.98) and chest discomfort (OR: 1.29, 95%CI 0.59–2.82, *p* = 0.52) did not differ between two groups (Fig. 4, Supplementary Fig. 5 [Additional file 1]).

We also did sensitive analyses including different study types, and the results were listed in Table 3. The digestive symptoms were more likely to appear in Azvudine group when the analysis was restricted in 3 cohort studies (OR: 4.84, 95% CI 0.85–27.7, *p* = 0.08), with high heterogeneity between studies. The differences were not significance in meta-analyses of total 6 studies (OR: 2.44, 95% CI 0.70–8.48, *p* = 0.16) and 3 RCTs (OR: 1.08, 95% CI 0.49–2.35, *p* = 0.85).

**Table 3:** Sensitivity analyses of adverse events based on study types

AE	RCTs				Cohort studies			
	No. of Studies	OR (95%CI)	*I* ^*2*^	*P*	No. of Studies	OR (95%CI)	*I* ^*2*^	*P*
Total AEs	3	0.80 (0.55–1.17)	9.5%	0.25	3	2.45 (0.73–8.21)	77%	0.15
Digestive system	3	1.08 (0.49–2.35)	15%	0.85	3	4.84 (0.85–27.7)	78%	0.08
Chest discomfort	3	0.51 (0.18–1.45)	0	0.86	1	NA		
Neurological system	2	NA			2	NA		
Elevated liver enzymes	2	NA			2	NA		
Elevated creatinine	1	NA			2	NA		

AE = adverse event; RCT = randomized controlled study; NA = no pooled analysis could be conducted because of the number of studies being less than 3

## Discussion

Azvudine was approved in emergency for fighting against COVID-19 since the epidemic of Omicron strain last winter in China [6]. The relationship of its administration and different clinical outcomes have been reported in several studies, without a determined conclusion. We therefore quantitatively review the efficacy and safety of this antinucleotide drug. To our knowledge, this is the first meta-analysis demonstrated that azvudine could reduce mortality risk in total patients. No significant associations were identified between azvudine and ICU admission, IMV/ECMO incidence, and rates of adverse events.

The importance of antiviral therapy was well recognized. Treating with specific antiviral drugs in time could help prevent the progression of COVID-19 to severe, or even critical state [30, 31]. Besides, recent meta-analyses found Paxlovid can reduce the mortality rate of COVID-19, compared with placebo, or no antiviral treatment [32, 33]. Similarly, our pooled analysis also showed reduced mortality risks in COVID-19 patients receiving azvudine, compared with those using placebo, or without specific antiviral therapies. This significant associations were also validated in patients with different disease severity, and patients older than 65 years old. The aforementioned results reinforced the necessity of using specific antiviral therapy, especially in patients over 65 years old, no matter the state of disease. Besides older age, COVID-19 patients with comorbidities were also in high risks of disease progression [34]. However, our meta-analysis did not identify survival benefit of using azvudine, in patients with hypertension, diabetes, or cardiovascular diseases. One possible explanation might be the statistical power was weakened due to decreased sample sizes and study numbers. And this might also explain why we did not identify associations between application of azvudine and ICU admission rates, or possibility of IMV/ECMO, compared with no antiviral therapies.

Head-to-head comparisons between azvudine and other antiviral drugs fighting against COVID-19 were not enough. We therefore could not employ quantitative pooled analysis, but summarized the results instead. Various studies showed azvudine could shorten the nucleic acid negative conversion time, but its efficacy seemed to be inferior to Paxlovid [9–11, 15, 24, 27]. However, the superiority of Paxlovid were not identified for mortality in COVID-19 patients. In addition, one study even found survival benefit in COVID-19 patients receiving azvudine, compared with those using Paxlovid [14]. Inactive viral debris that has lost pathogenicity may also lead to nucleic acid positivity [35]. This could partly explain the inconsistency of different outcomes.

A key concern for Paxlovid administration was its effect on cytochrome system and the resulting drug interactions [36]. Azvudine therefore had its advantages in patients with comorbidities with combined therapies. Future studies comparing the efficacy of these two drugs in fighting against COVID-19, especially in specific populations are still needed, to better illustrate whether azvudine could be an alternative choice, or just a supplementation to Paxlovid, and the suitable scenarios for its application.

The safety of azvudine has previously been demonstrated in the treatment of acquired immune deficiency syndrome [37]. As a nucleoside analogue, its liver and kidney impairment are a matter of concern. Although occasionally reported, we did not identify safety concerns of azvudine in relevant cohort studies. Similarly, our pooled analysis showed that the total adverse event rates of azvudine was similar to that of the control group, which was consistent with the findings of a previously published meta-analysis [16]. Besides, there was no significant difference between the two groups in the incidence of gastrointestinal symptoms, neurological symptoms and elevation of liver enzymes and creatinine. These evidences indicated that azvudine was safe for the treatment of COVID-19 patients.

There are some limitations in our meta-analysis. Firstly, most studies included for meta-analysis were retrospective studies. We tried to reduce the risk of bias by choosing results calculated after propensity score matching or multivariable adjustment. Second, heterogeneity was showed in the analysis of mortality risks in patients with cardiovascular disease, as well as the analysis of adverse events. Publication bias was also existed in studies mentioning patients with cardiovascular diseases. We therefore employed random-effect model for these analyses. In addition, we performed sensitive analyses for adverse events, and the results remained when only RCTs included for reduced heterogeneity. In summary, the pooled results should be interpreted with caution and further validated in future by enrolling high-quality prospective studies.

## Conclusions

The current meta-analysis found azvudine was effective in reducing mortality risks of COVID-19 patients, compared with no antiviral therapies, especially in patients older than 65 years, no matter the severity of disease. Meanwhile, the incidence of adverse events of patients using azvudine was comparable to those with no antiviral treatment. High-quality prospective studies are required to confirm our findings. We also expect future evidences centered on comparison of azvudine and other antiviral drugs, and application of azvudine in specific populations.

### Electronic supplementary material

Below is the link to the electronic supplementary material.

**Supplementary Material 1: Supplementary Table 1**. Search strategy and original results from database. **Supplementary Table 2**. Risk of bias of included cohort studies. **Supplementary Table 3**. Risk of bias of included randomized controlled studies. **Supplementary Fig. 1**. Publication bias evaluated by funnel plots for efficacy of azvudine. **Supplementary Fig. 2**. Publication bias evaluated by funnel plots for adverse events of azvudine. **Supplementary Fig. 3**. Risk of bias of included studies. **Supplementary Fig. 4**. Effectiveness of azvudine on mortality in COVID-19 patients. **Supplementary Fig. 5**. Adverse events of azvudine compared with controls

## Data Availability

No datasets were generated or analysed during the current study.

## Abbreviations

AE: Adverse event
CI: Confidence interval
COVID-19: Coronavirus disease 2019
L.OVE: Living Overview of Evidence
FNC: Azvudine
ECMO: Extracorporeal membrane oxygenation
HR: Hazards ratio
ICU: Intensive care unit
IMV: Invasive mechanical ventilation
L.OVE: Living Overview of Evidence
OR: Odds radio
Paxlovid: Nirmatrelvir/Ritonavir
RCTs: Randomized controlled studies
SARS-CoV-2: Severe acute respiratory syndrome coronavirus-2
R: Retrospective cohort
RN: Ritonavir-Nirmatrelvir
*T*_FNANC_: Time to first nucleic-acid negative conversion
NA: Not available
