# Supplementary material for: Effectiveness of azvudine in reducing mortality of COVID-19 patients: a systematic review and meta-analysis
Source: Virol J. 2024 Feb 23;21:46. doi: 10.1186/s12985-024-02316-y (PMC10893615; doi:10.1186/s12985-024-02316-y)
Supplement: Supplementary file 1 — Supplementary Material 1: Supplementary Table 1. Search strategy and original results from database. Supplementary Table 2. Risk of bias of included cohort studies. Supplementary Table 3. Risk of bias of included randomized controlled studies. Supplementary Fig. 1. Publication bias evaluated by funnel plots for efficacy of azvudine. Supplementary Fig. 2. Publication bias evaluated by funnel plots for adverse events of azvudine. Supplementary Fig. 3. Risk of bias of included studies. Supplementary Fig. 4. Effectiveness of azvudine on mortality in COVID-19 patients. Supplementary Fig. 5. Adverse events of azvudine compared with controls [file 12985_2024_2316_MOESM1_ESM.pdf]

Supplementary Table 1. Search strategy and original results from database.

		Pubmed	Embase	Cochrane	Web of Science	L.OVE*
#1	Azvudine	42	79	22	65	45
#2	FNC	545	699	52	909	8
#3	2′-deoxy-2′-β -fluoro-4′azidocytidine	45	2	0	0	0
#4	COVID-19[MeSH Terms]	237,193	420,415	16,585	413,378	
#5	SARS-CoV-2[MeSH Terms]	158,358	155,355	6,449	242,537	
#6	Coronavirus Disease 2019	367,890	347,358	7,317	153,092	
#7	2019 nCoV Disease	366,542	30	179	5198	
#8	2019 nCoV Infection	366,572	200	184	5024	
#9	2019 Novel Coronavirus Disease	366,545	484	688	17,559	
#10	2019 Novel Coronavirus Infection	366,561	91	390	15,450	
#11	SARS-CoV-2 Infection	367,959	34,477	3,804	171,635	
#12	Severe Acute Respiratory Syndrome Coronavirus 2	210,652	107,630	2,477	138,435	
#13	SARS Coronavirus 2	208,574	7,655	4,249	175,645	
#14	#1 OR #2 OR #3	568	827	63	953	45
#15	#4 OR #5 OR #6 OR #7 OR #8 OR #9 OR #10 OR #11 OR #12 OR #13	383,387	451,333	17,933	455,690	
#16	#14 AND #15	27	64	18	30	45

* L.OVE: Epistemonikos COVID-19 Living Overview of Evidence database

Supplementary Table 2. Risk of bias of included cohort studies.

Author	Year	Bias due to confounding	Bias in selection of participants into study	Bias in classification of interventions	Bias due to deviations from intended intervention	Bias due to missing data	Bias in measurement of outcomes	Bias in selection of the reported result	Overall
Wenmei Chen	2023	Moderate	Low	Low	Low	Low	Low	Moderate	Moderate
Yating Dian	2023	Moderate	Low	Low	Low	Low	Low	Moderate	Moderate
Yuan Gao	2023	Moderate	Low	Low	Low	Low	Low	Moderate	Moderate
Jiasheng Shao	2023	Serious	Low	Low	Low	Low	Low	Moderate	Serious
Yuming Sun	2023	Moderate	Low	Low	Low	Low	Low	Moderate	Moderate
Hui Yang	2023	Moderate	Low	Low	Low	Low	Low	Moderate	Moderate
Kaican Zong	2023	Moderate	Low	Low	Low	Low	Low	Moderate	Moderate
Minxue Shen	2023	Moderate	Low	Low	Low	Low	Low	Moderate	Moderate
Xinjie Han	2023	Moderate	Low	Low	Low	Low	Low	Moderate	Moderate
Ru Chen	2023	Moderate	Low	Low	Low	Low	Low	Moderate	Moderate
Yiling Zhou	2023	Unclear	Low	Low	Low	Low	Low	Moderate	Unclear
Shunlai Shang	2023	Moderate	Low	Low	Low	Low	Low	Moderate	Moderate
Qinqin Zhao	2023	Moderate	Low	Low	Low	Low	Low	Moderate	Moderate

Guangtong Deng	2023	Moderate	Low	Low	Low	Low	Low	Moderate	Moderate

Supplementary Table 3. Risk of bias of included randomized controlled studies.

Autor	Year	Random sequence generation	Allocation concealment	Blinding of participants and personnel	Blinding of outcome assessment	Incomplete outcome data	Selecting reporting	Other bias
Zhigang Ren	2020	Low	Unclear	High	Low	Low	Low	Low
Renato Martins da Silva	2023	Low	Low	Unclear	Unclear	Low	Low	Low
Paula Cabral	2022	Low	Low	Unclear	Unclear	Low	Low	Low

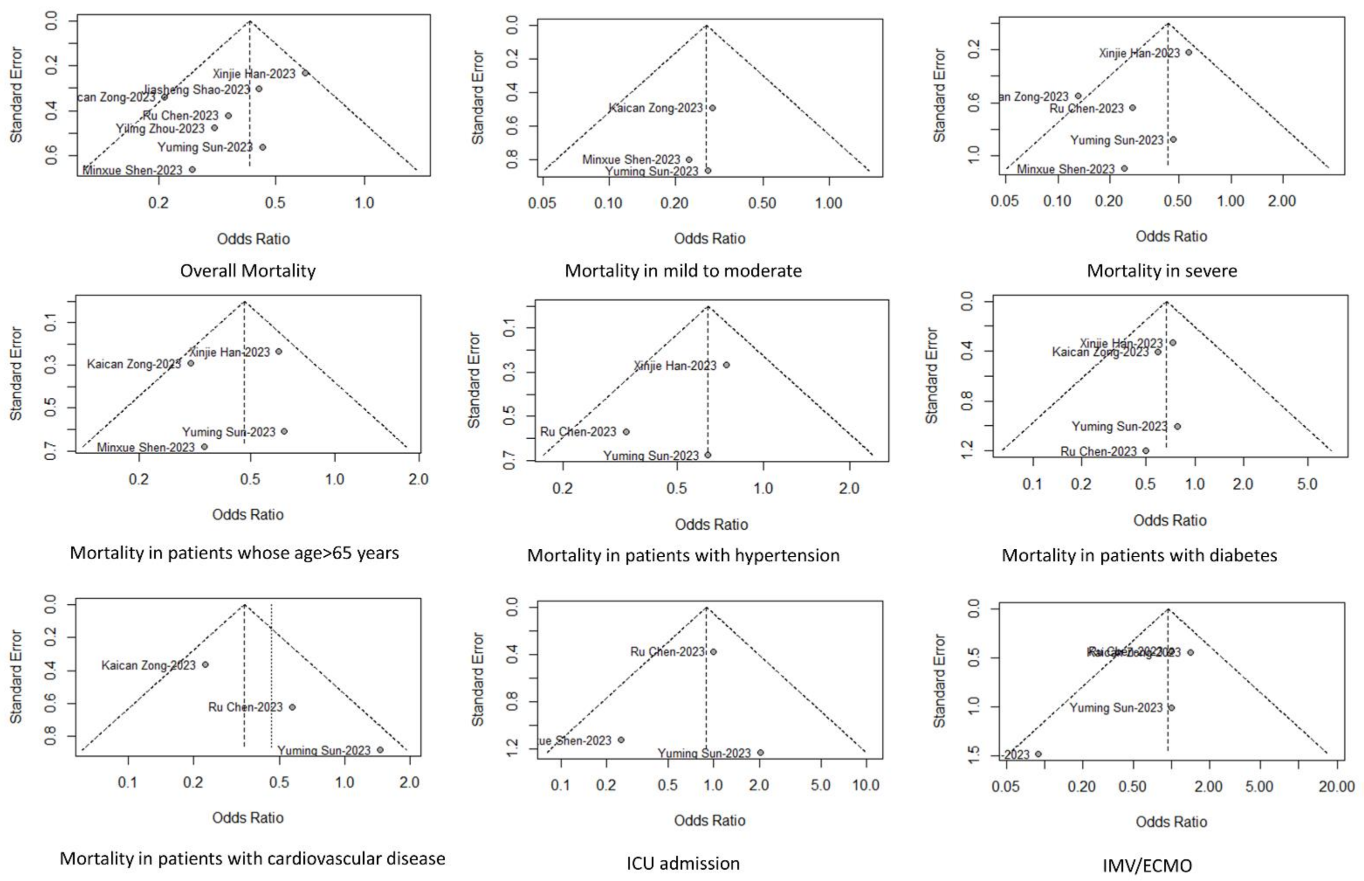

Supplementary Fig. 1. Publication bias evaluated by funnel plots for efficacy of azvudine.

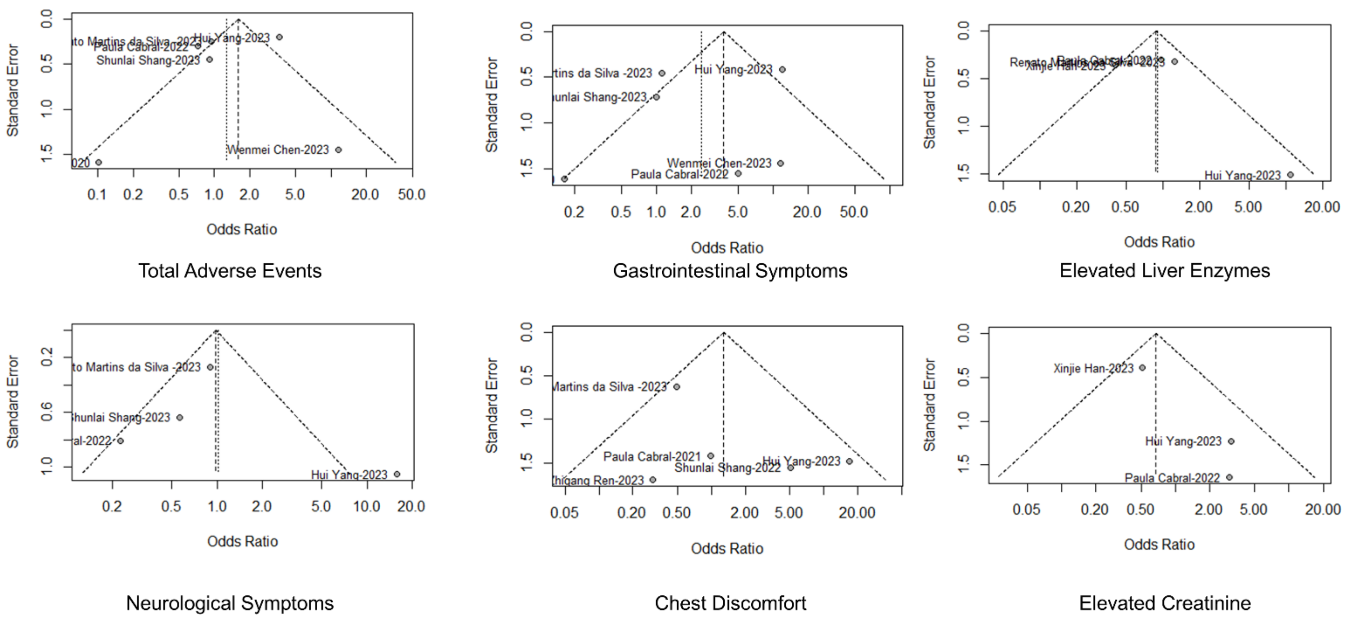

Supplementary Fig. 2. Publication bias evaluated by funnel plots for adverse events of azvudine.

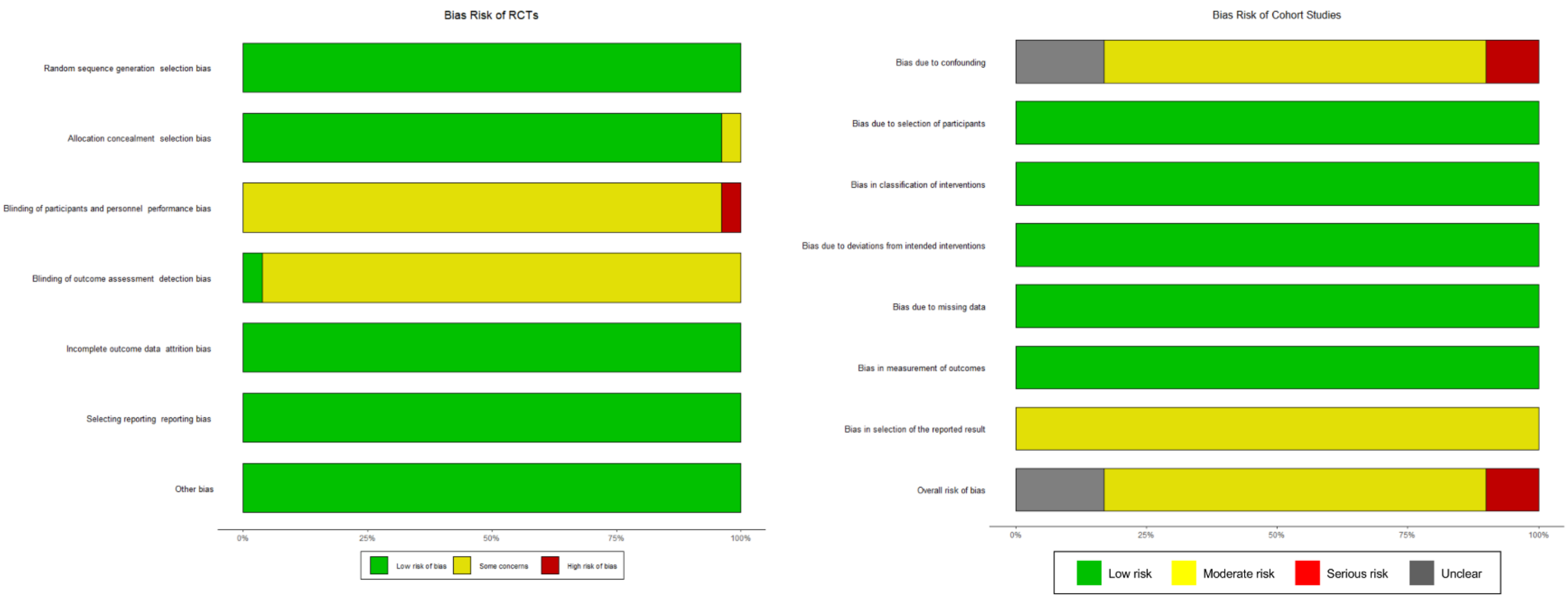

Supplementary Fig. 3. Risk of bias of included studies.

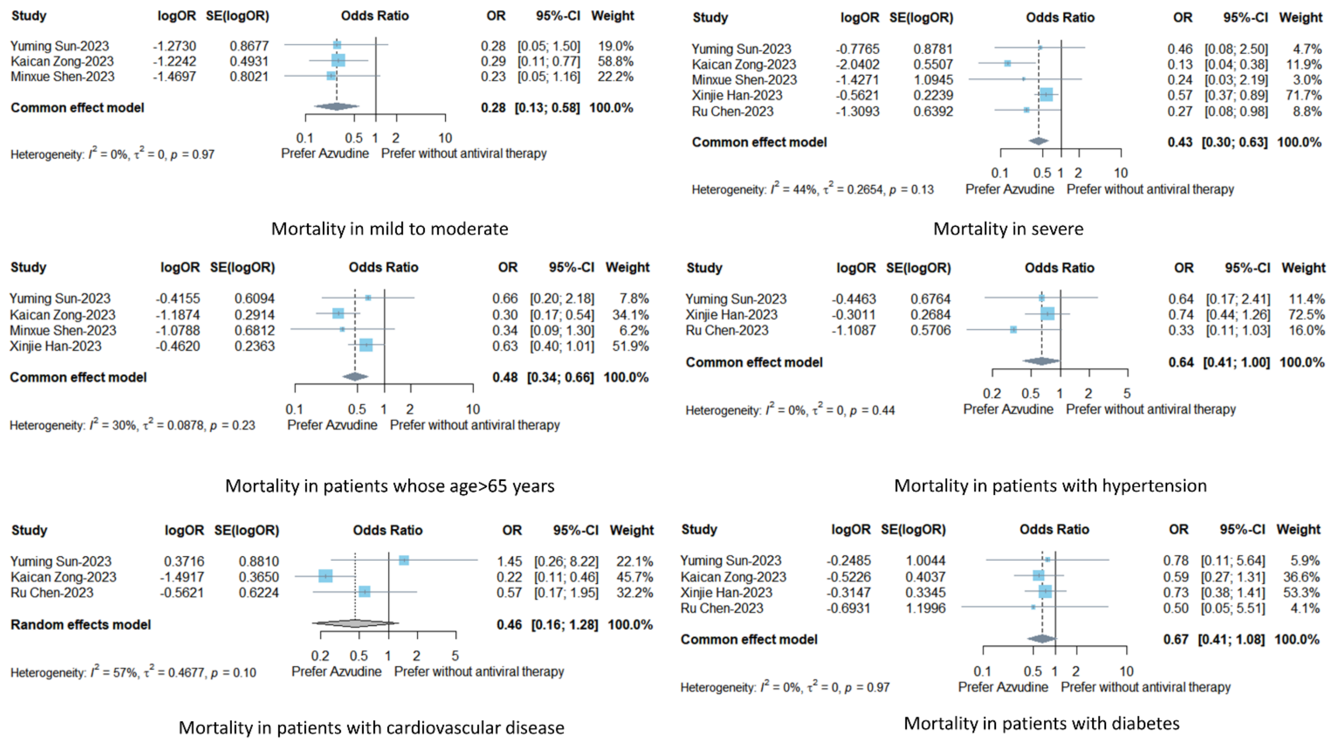

Supplementary Fig. 4. Effectiveness of azvudine on mortality in COVID-19 patients.

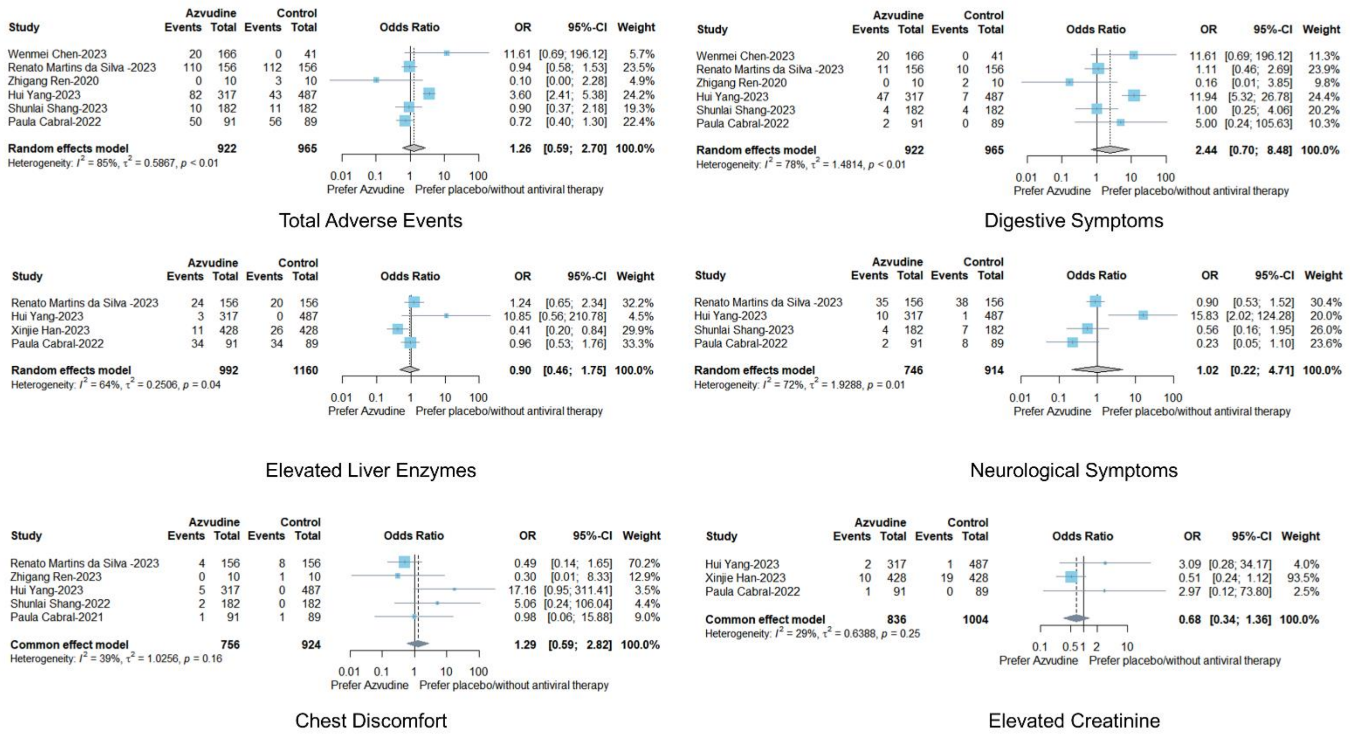

Supplementary Fig. 5. Adverse events of azvudine compared with controls.
